# Tick-fever1Read at the December meeting of the Veterinary Medical Society of the Ontario Veterinary College.

**Published:** 1897-06

**Authors:** D. F. Luckey

**Affiliations:** Veterinary Student, Ontario Veterinary College


### TICK-FEVER.[1]

By D. F. Luckey,
VETERINARY STUDENT, ONTARIO VETERINARY COLLEGE.

One of the most common diseases among cattle may be appropriately called tick-fever. This disease is known among laymen by a number of different names, such as dry murrain, bloody murrain, red-water, Texas-fever, Southern cattle-fever, Spanish fever, etc.

Tick-fever is caused, in susceptible cattle, by a tick, *Boophilus bovis*. The tick, in biting the cow, injects an animal microörganism (*Pyrosoma bigeminum*) into her blood. Once in the blood of a susceptible animal, this microörganism propagates at an enormous rate, and in a few days will produce death.

Where the ticks get the microörganism of this disease, and exactly how they inject it into the cow's blood, are not known. It is sufficient to say here that when native, Northern, or susceptible cattle are exposed to this tick, the disease invariably results. It does not matter whether the cattle be moved to the ticky range or the ticks be voluntarily carried to and placed upon the cattle; the result is the same.

Tick-fever has existed in the United States ever since the cattle

[1] Read at the December meeting of the Veterinary Medical Society of the Ontario Veterinary College.

of the early Spanish and English settlers began to mingle together. It is safe to say that the ticks originated with the Spanish cattle. They are now confined to the Southern States, and quarantine regulations guard against their being spread broadcast throughout the Northern States during the warm season of each year. They are unable to survive the cold winters of the Northern States, so that when Northern territory accidentally becomes infested the infection will last only till the cold weather of the following winter. The warm climate of the Southern States tolerates their existence throughout the year.

The question naturally arises, Why do the Southern cattle, which carry the fever-tick almost constantly, not suffer from the disease? The answer is that they are immune. It is a well-known fact that one attack of many diseases results in subsequent immunity. Dr. Sternberg's theory of immunity, applied to tick-fever, would indicate that there exists in the serum of the blood of Southern unsusceptible or immune cattle an antitoxin of this particular disease-germ, and that the germ is destroyed by the antitoxin as soon as it enters the circulation. If the germ were not destroyed, and the only action of the antitoxin were to counteract the toxic products, there would be a constant state of pernicious anæmia, as the pathology of this disease shows to exist when the germ is active. There are reasons to believe that immunity is secured by the calf undergoing an attack of this fever while quite young, and that such immunity is permanent. Such an attack is very mild: in fact, hardly perceptible to laymen. Breeders of thoroughbred cattle say that native calves shipped to infested territory usually do well, while yearlings to adults shipped to the same pastures almost invariably die. It is my opinion that native calves could be prepared for Southern breeders by inoculating them with the ticks at from two to four months of age. The indications are that an attack in the adult does very little toward securing immunity for the animal.

*Symptoms.* External symptoms of tick-fever are cessation of rumination, anorexia, digestive derangement, drooping ears, high pulse and temperature, and unconcerned about surroundings. An attempt to walk will show a swaggering gait and indications of weakness. After the first day or two the urine will be red. If the thermometer is at hand, the peculiarly intermittent fever of this disease can be discovered. The temperature of the first day will be about normal in the forenoon and 105° to 107° in the afternoon. The temperature of each successive forenoon is a little higher until about the fifth or sixth day, when the morning temperature is the same as the evening. But a scant escape of blood will take place from an incision through the skin. The presence of the tick, with the above symptoms, is reasonable proof of tick-fever, yet a microscopic examination of the blood to find the disease-microbe is the only absolute test.

*Pathology.* Tick-fever is strictly a blood-disease. Constipation, impaction, dysentery, nephritis, engorgement of the spleen, derangement of the liver, and congestion of the lungs are simply incidental, and do not exist until produced by changes which take place during the course of the disease. The germ takes up its abode in the red blood-corpuscles and destroys them. The reduction in four to six days is estimated to be from three-fourths to five-sixths of the normal number, leaving the blood in a wonderfully depleted condition. I believe that death results directly from the lack of oxygen in the system. The débris of the broken-down corpuscles at once engorges the spleen, clogs up the small bloodvessels of the liver, causing disintegration, and often causes congestion of the lungs and inflammation of the kidneys. The toxic products probably cause the fever.

*Post-mortem.* A post-mortem examination shows an enlarged spleen, diseased condition of the liver, giving it a lighter brown hue. There are often signs of inflammation of the kidneys and congestion of the lungs, and nearly always a derangement of the digestive tract. There is either constipation, with impaction of the manifold, or inflammation of the intestines from excessive diarrhœa. A microscopic examination of the blood shows a paucity of red corpuscles.

*Treatment.* Protective treatment consists in keeping the ticks off of the cattle. Directions concerning protective treatment would make up a long essay, and cannot be taken up here.

I give the following as the medicinal treatment I think best for an animal affected with tick-fever: Withhold all dry food. Tempt the appetite with bran-slops, green grass or corn, apples, water-melons, or anything of the kind that may be at hand. I think such food tends to prevent impaction and constipation, and may be beneficial to digestion. Salt should be given in moderate quantities, as it stimulates the appetite for water. Plenty of pure, cold water should be allowed. To be successful, the treatment must begin as soon as the disease manifests itself. It will be useless if delayed until the resulting changes have taken place. If a purgative is indicated, give linseed oil, a pint and a half. It is well sometimes to use a febrifuge, the tincture of aconite, thirty drops every four hours, being probably the best.

The treatment should be directed principally to the destruction of the germ in the blood at an early stage of the disease. Quinine sulphate is the best known medicine for this purpose. Give three drachms every three hours until two to five ounces have been taken, and if properly absorbed it will produce valuable results. If the manifold has become dried and impacted, and there is constipation, quinine given internally will be of no value whatever. The use of two-drachm doses of quinine bisulphate intraveneously every three hours for a day is a treatment that deserves further trial.

After recovery allow the animal plenty of green food and give a blood tonic.

